# A Simulation Model of Periarterial Clearance of Amyloid-β from the Brain

**DOI:** 10.3389/fnagi.2016.00018

**Published:** 2016-02-12

**Authors:** Alexandra K. Diem, Mingyi Tan, Neil W. Bressloff, Cheryl Hawkes, Alan W. J. Morris, Roy O. Weller, Roxana O. Carare

**Affiliations:** ^1^Institute for Complex Systems Simulation, School of Electronics and Computer Science, University of SouthamptonSouthampton, UK; ^2^Computational Engineering and Design, Faculty of Engineering and the Environment, University of SouthamptonSouthampton, UK; ^3^Fluid Structure Interactions, Faculty of Engineering and the Environment, University of SouthamptonSouthampton, UK; ^4^Clinical and Experimental Sciences, Faculty of Medicine, University of SouthamptonSouthampton, UK; ^5^Institute for Life Sciences, University of SouthamptonSouthampton, UK

**Keywords:** Alzheimer's disease, brain, cerebral amyloid angiopathy, dextran, diffusion, lymphatic drainage, perivascular drainage, simulation model

## Abstract

The accumulation of soluble and insoluble amyloid-β (Aβ) in the brain indicates failure of elimination of Aβ from the brain with age and Alzheimer's disease (AD). There is a variety of mechanisms for elimination of Aβ from the brain. They include the action of microglia and enzymes together with receptor-mediated absorption of Aβ into the blood and periarterial lymphatic drainage of Aβ. Although the brain possesses no conventional lymphatics, experimental studies have shown that fluid and solutes, such as Aβ, are eliminated from the brain along 100 nm wide basement membranes in the walls of cerebral capillaries and arteries. This lymphatic drainage pathway is reflected in the deposition of Aβ in the walls of human arteries with age and AD as cerebral amyloid angiopathy (CAA). Initially, Aβ diffuses through the extracellular spaces of gray matter in the brain and then enters basement membranes in capillaries and arteries to flow out of the brain. Although diffusion through the extracellular spaces of the brain has been well characterized, the exact mechanism whereby perivascular elimination of Aβ occurs has not been resolved. Here we use a computational model to describe the process of periarterial drainage in the context of diffusion in the brain, demonstrating that periarterial drainage along basement membranes is very rapid compared with diffusion. Our results are a validation of experimental data and are significant in the context of failure of periarterial drainage as a mechanism underlying the pathogenesis of AD as well as complications associated with its immunotherapy.

## 1. Introduction

Alzheimer's disease (AD) is characterized pathologically by the accumulation of soluble and insoluble amyloid-β (Aβ) in the extracellular spaces of the brain and tau-related neurofibrillary tangles within neurons (Duyckaerts and Dickinson, 2011). Insoluble Aβ forms plaques within cerebral gray matter and also accumulates within the walls of cerebral capillaries and arteries as cerebral amyloid angiopathy (CAA; Weller et al., 2008). The increased levels of both soluble and insoluble Aβ in the brain suggest that failure of elimination of Aβ is a significant factor in the pathogenesis of AD (Carare et al., 2013; Weller et al., 2015). There is a variety of known mechanisms for the elimination of Aβ from the brain, including degradation of insoluble plaques of Aβ by microglia and the degradation of Aβ by enzymes such as neprilysin (Miners et al., 2011). Aβ is absorbed into the blood by receptor-mediated mechanisms (Zlokovic, 2004), passes into the cerebrospinal fluid (CSF) (Iliff et al., 2012, 2013) and also drains out of the brain along the pericapillary and periarterial lymphatic drainage pathways (Carare et al., 2013). There is evidence for failure of several mechanisms of elimination of Aβ with age and in AD including failure of lymphatic drainage (Carare et al., 2013).

The brain does not possess the conventional lymphatic drainage vessels that are present in most organs of the body. Furthermore, although Aβ is produced by most cells in the body, there is no apparent failure of elimination of soluble Aβ in most organs except the brain. Despite the lack of defined lymphatic vessels, there is a well-developed and efficient lymphatic drainage of interstitial fluid ISF and soluble metabolites, including Aβ, from the brain (Szentistvanyi et al., 1984; Carare et al., 2008, 2013) but lymphatic drainage is impaired with age (Hawkes et al., 2011; Weller et al., 2015). Experimental studies have shown that when soluble tracers including Aβ are injected into cerebral gray matter, they initially diffuse through the extracellular spaces, enter basement membranes around cerebral capillaries and then flow out of the brain along basement membranes between smooth muscle cells in the tunica media of cerebral arteries (Carare et al., 2008; Hawkes et al., 2011). Such periarterial lymphatic drainage is rapid and when drainage of tracer is complete, the route appears to be outlined by perivascular macrophages that have imbibed tracer during its passage along the periarterial lymphatic drainage pathway (Cserr and Ostrach, 1974; Cserr et al., 1977, 1981; Carare et al., 2008).

**Figure 1:**
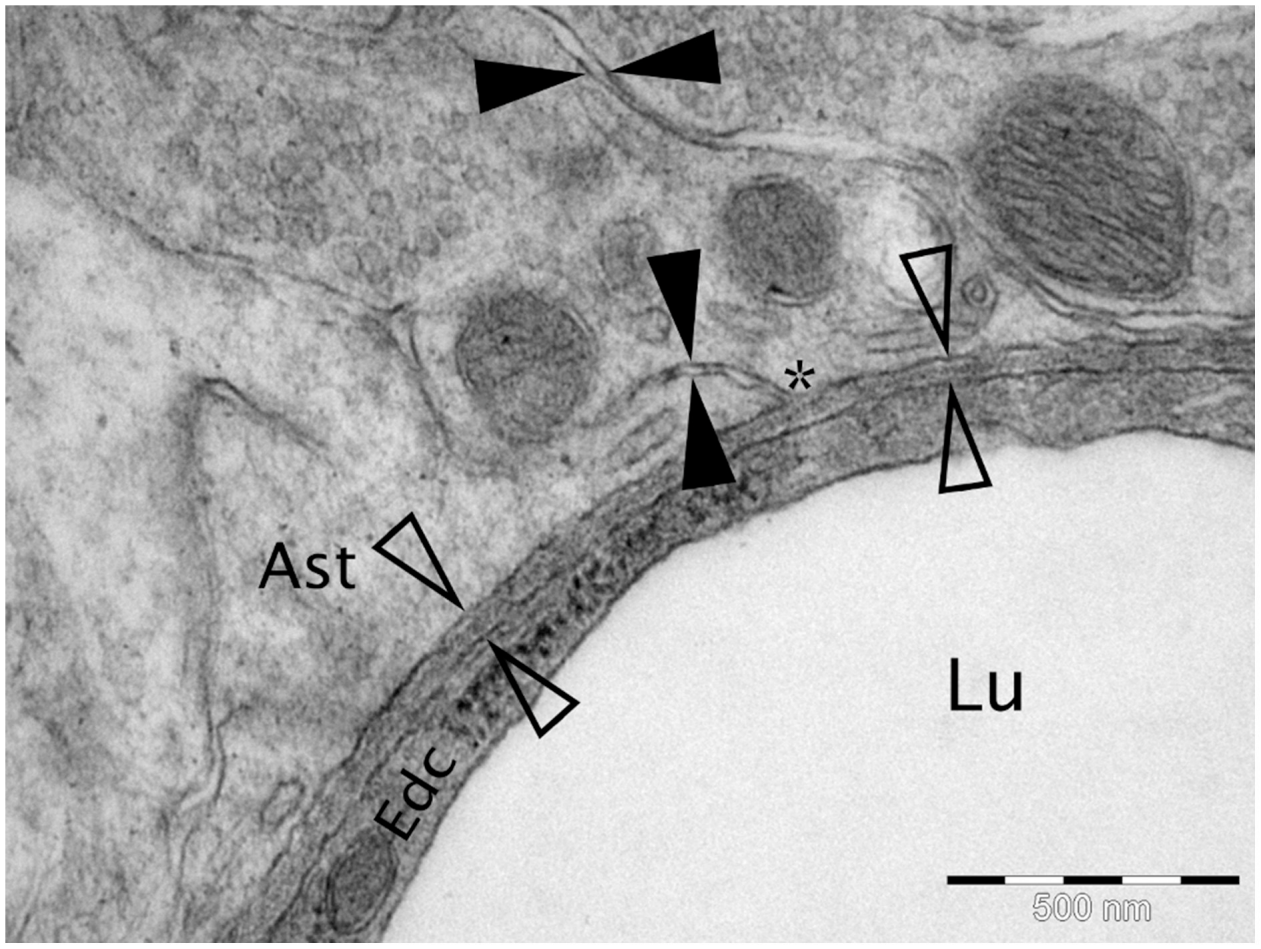
**Micrograph of cortical Wistar Kyoto rat capillary**. The cerebrovascular basement membrane is the electron dense area between the four empty arrow heads. The extracellular matrix is the area between the filled arrow heads. The ^*^ marks an area of continuity between the extracellular matrix and the cerebrovascular basement membrane. Lu Capillary Lumen, Edc - Endothelial cell, and Ast - Astrocyte end foot. TEM, 50,000x, scale bar 500 nm.

Soluble metabolites, produced by cells within the central nervous system (CNS), begin their journey out of the brain by entering interstitial fluid ISF and passing through the narrow ECS in the brain before entering the perivascular drainage pathways within basement membranes (100–150 nm thick) in the walls of capillaries and arteries (Carare et al., 2008). This transport mechanism is mirrored by the deposition of Aβ in basement membranes in AD. The narrow gaps that separate cells in the gray matter of the CNS form the ECS that are in direct continuity with basement membranes of capillaries (Figure 1). Recent data demonstrate that the ECS in the cerebral cortex is 38–68 nm wide, which is significantly larger than the traditional value of 20 nm observed by electron microscopy (Thorne and Nicholson, 2006). Functionally, the ECS provides a pathway for diffusion and exchange of ions and molecules between cells. In the brain, the space between the cells is occupied by ISF derived from the blood and CSF. Water generated as a result of oxidation of glucose to CO2 and water could provide a 10 % contribution to the total volume of ISF (Abbott, 2004). A large fraction of ISF may be derived from the blood through the capillary endothelium, driven by Na^+^-K^+^-ATPase, with water following passively (Abbott, 2004).

Diffusion of molecules through the extracellular spaces of cerebral gray matter is well characterized. The most widely used method for studying diffusion is the real-time iontophoretic method TMA (tetramethylammonium) devised by Nicholson and Phillips, demonstrating that molecules execute random walks through the extracellular space (Nicholson et al., 2000). However, there is a gap in our understanding of the dynamics of periarterial drainage of ISF and solutes, including Aβ, out of the brain and how these dynamics relate to diffusion. Understanding such dynamics is important not only for clarifying the pathogenesis of AD and designing therapies, but also for characterizing drug delivery to the brain and spinal cord (Carare et al., 2013).

In previous experiments, it has been shown that, following injection into the central gray matter of the mouse brain, 4 kDa fluorescent tracer spreads diffusely through the brain parenchyma and enters basement membranes of cerebral capillaries and cerebral arteries (Carare et al., 2008). Within 5 min of the injection, tracer is present at the surface of the brain (Carare et al., 2008). The question we seek to answer here is whether progress of the tracer from the injection site to the surface of the brain is by diffusion or by another mechanism along capillary and periarterial pathways. In order to resolve this question in the present paper, we use a computational model to define the process of periarterial drainage in the context of diffusion within the extracellular spaces of cerebral gray matter. First, we construct a mathematical computational model that encompasses both diffusion and periarterial lymphatic drainage and second we simulate the movement of solutes of low molecular weight, similar to Aβ, through the extracellular spaces of gray matter in the brain. By these techniques, we demonstrate that diffusion through the extracellular spaces cannot alone account for the rapid movement of solutes toward the surface of the brain, but, when coupled with periarterial drainage, solutes reach the walls of arteries on the surface of the brain within the observed time-scale of 5 min. While similar questions have been addressed in the literature, our study is the first to specifically address the issue of perivascular drainage along basement membranes. Specifically, work by Groothuis et al. (2007) emphasizes the difference in clearance rates of various tracers, dependent on diffusion, convection, and transport across the endothelium into the blood, but the time scales analyzed were over several hours, whereas in our study we address the rapid entry of solutes into the basement membranes. Our findings help to clarify the dynamics of lymphatic drainage of the brain that plays a significant role in the elimination of Aβ and its failure in AD.

## 2. Methods

**Figure 2:**
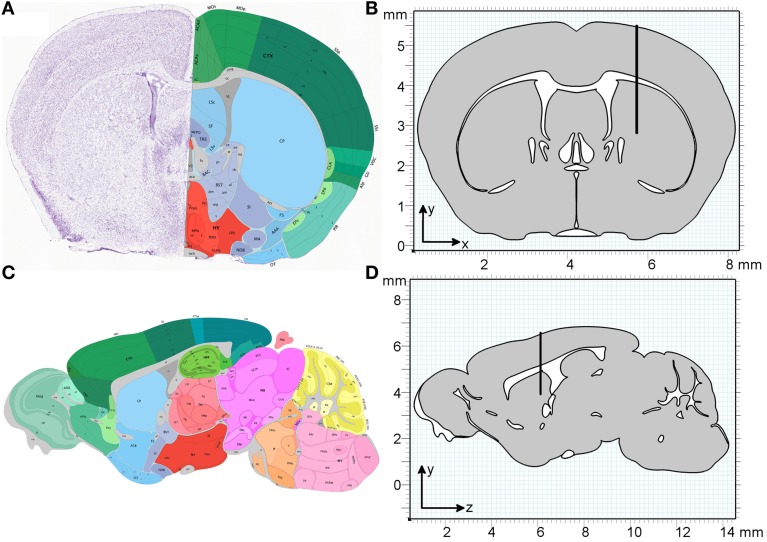
**Geometry of the coronal and sagittal sections of a mouse brain used in the simulations. (A)** Coronal slice as obtained from the Allen Institute for Brain Science (Lein et al., 2007). **(B)** The homogenized simulation geometry of the coronal slice has a width of 8 mm and a height of 5.5 mm. The thin black rectangle depicts the position of the injection needle, with the tip at position (5.7, 2.8 mm). **(C)** Sagittal slice as obtained from the Allen Institute for Brain Science (Lein et al., 2007). **(D)** The homogenized simulation geometry of the sagittal slice has a length of 13.7 mm and a height of 5.5 mm. The black rectangle depicts the position of the injection needle, with the tip at position (6.1, 3.9 mm). One material type is defined for all gray matter (gray) and one material type is defined for all white matter (white).

All simulations are carried out using the “Transport of Diluted Species” module in COMSOL Multiphysics 4.4 (build 248) using a time-dependent solver over 32 min. This period was divided into 2 min for the injection of molecules and 30 min observation time. The geometries used are a two-dimensional (2D) coronal and a 2D sagittal section from the Allen Brain Atlas (Mouse Brain Reference Atlas, Lein et al., 2007). The slices were chosen according to the injection site in Carare et al. (2008) (bregma 1 mm anterior, lateral + 1.5 mm, 2.5 mm deep) and were estimated as slice 55 (coronal) and slice 14 (sagittal). Both sections were simplified by homogenizing all gray and white matter, i.e., defining one averaged material for all gray matter and one averaged material for all white matter, where all sections that are not listed as “gray” in the atlas were interpreted as white matter. Figure 2 compares the slices as obtained from the Allen Brain Atlas and the geometries used in the simulations. The geometries were created using the MATLAB LiveLink for COMSOL. For each slice two separate geometries (gray and white matter) were created, which were combined into a single geometry in COMSOL (Figure 2). To simulate the injection needle a rectangle with a width of 50 µm and a length of 2.5 mm was inserted 1.5 mm to the right of the vertical centre line in the coronal slice and 5.5 mm from the front of the brain in the sagittal slice. The geometry provides the basis for the creation of a mesh of elements on which chemical species concentrations are evaluated during the simulation.

### 2.1. Mesh size and time stepping

**Figure 3:**
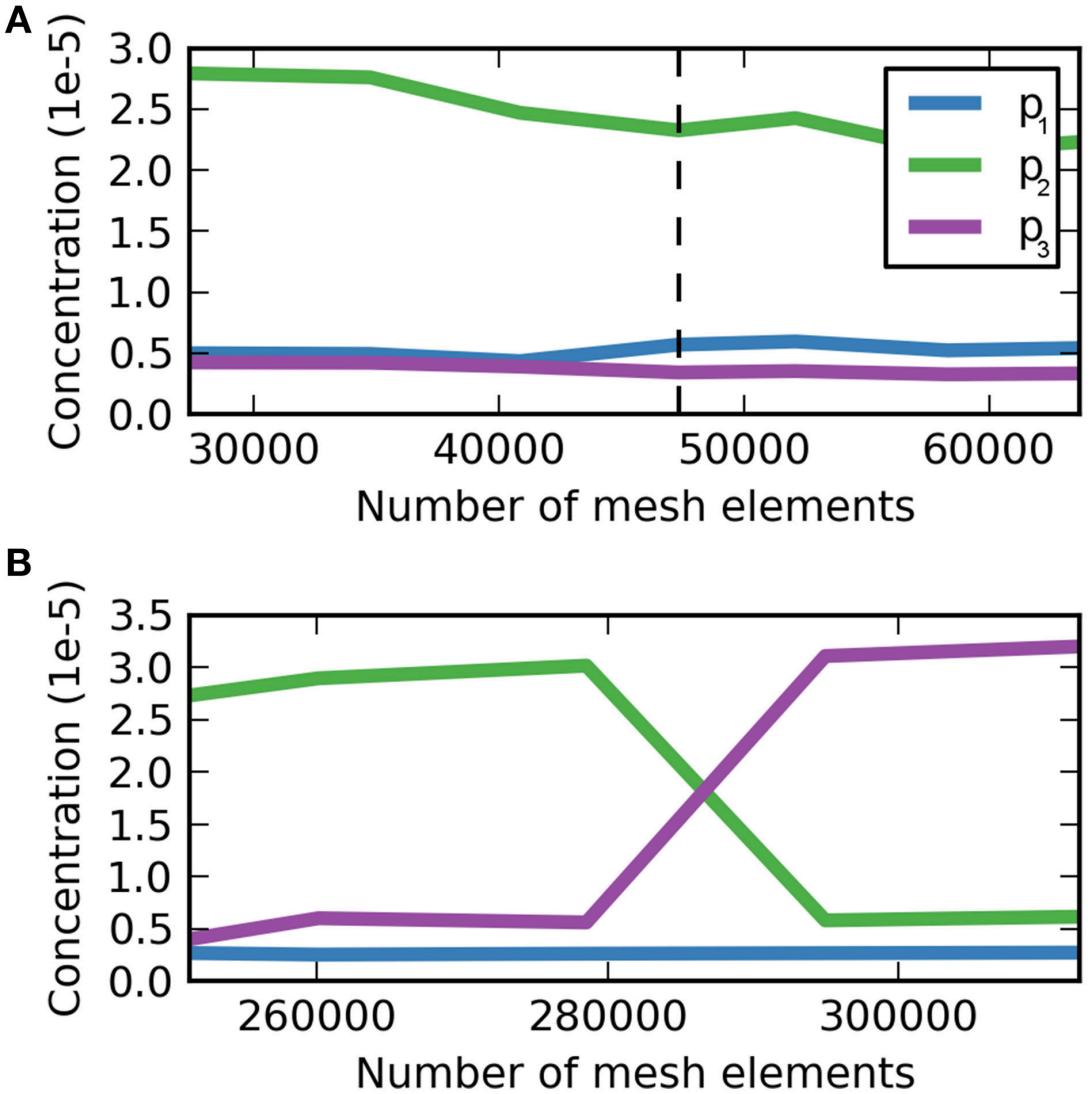
**Dimensionless dextran concentration *c* over the number of mesh elements at 5 min after the injection for three different locations. (A)** Mesh convergence study for the coronal slice, p_1_ = (5.7, 2.8 mm), p_2_ = (6.0, 2.8 mm), p_3_ = (6.3, 2.8 mm). The dashed vertical line indicates the optimal mesh, which consists of 47,342 triangular mesh elements. At this size *c* is fully converged for p_3_, but not for p_1_ and p_2_, where *c* oscillates around a value similar to that at the chosen mesh size with the magnitude of oscillations much smaller than the detection limit. **(B)** Mesh convergence study for the sagittal slice. **(B)** Mesh convergence study for the sagittal slice, p_1_ = (6.1, 3.9 mm), p_2_ = (6.4, 3.9 mm), p_3_ = (6.7, 3.9 mm). The optimal mesh was found at a size of 294,967, where all concentration values have reached convergence.

Following the setup of the model simulation case A1 (refer to the remainder of this section for implementation details) was used to carry out a mesh convergence study on the coronal slice to ensure that the choice of mesh resolution does not alter the results. The mesh convergence study was based on the “Adaptive mesh refinement” option in the COMSOL solver. This option adapts the mesh size according to an error indicator ε, which in this case was chosen as the root mean square of the derivative of dextran concentration *c* in *x* and *y* and *y* and *z* direction in the coronal and sagittal slice, respectively. For the coronal slice we define

(1) $$\varepsilon_{\text{coronal}}=\sqrt{{\left(\frac{dc}{dx}\right)}^{2}+{\left(\frac{dc}{dy}\right)}^{2}}$$

and analogously for the sagittal slice. The “adaptive mesh refinement” option has the advantage of automatically refining regions where ε is high. The convergence criterion for evaluating the meshes that were created by COMSOL the concentration *c* of dextran in the ECS at three different locations at 5 min after the injection was used (Figure 3). The concentration was dedimensionalized using the injection concentration of 0.33 mM. For the coronal slice a mesh containing 47,342 triangular mesh elements was deemed optimal, while for the sagittal the optimal mesh contains 294,967 triangular mesh elements. Note that the sagittal slice is much larger than the coronal slice and therefore it contains more mesh elements.

The optimal time step for the simulations was determined using the Courant-Friedrichs-Lewy (CFL) condition, where the Courant number

(2) $$C=\frac{u\cdot \Delta t}{\Delta x}$$

is smaller than some maximum number C_max_, chosen to be 1. Then Δ*x* represents the side length of the smallest triangular mesh element and *u* represents the flow velocity. With Δ*x* = 2.36·10^−6^*m*, *u* = 8.33·10^−6^*ms*^−1^, and choosing *C* = 0.5 we obtain Δ*t* = 0.14*s*.

### 2.2. Diffusion and bulk flow

The transport of a diluted substance such as dextran via diffusion and convective forces can be described by writing

(3) $$\frac{\partial c}{\partial t}=D^{*}\nabla^{2}c-(u\cdot \nabla )c-kc,$$

where *c* is the concentration of the substance within the ECS in moll^−1^ or M, ***u*** is the convective velocity vector in m s^−1^, *k* is the uptake rate in s^−1^ and D^*^ is the effective diffusion coefficient in m^2^ s^−1^ (Nicholson, 2001). It is important to note at this point that although the simulations are carried out in a 2D plane the concentration units are the same as for a three-dimensional (3D) simulation. COMSOL implicitly assumes a third dimension in the out of plane direction with a unit depth of 1 m for all 2D simulations. The effective diffusion coefficient D^*^ of a substance is lower than its diffusion coefficient *D* in a free medium due to the tortuosity of the diffusion pathways in the medium. The tortuosity of a medium is described via the parameter λ and we obtain D^*^ by writing D^*^ = *D*/λ^2^. The factors contributing to the tortuosity and the techniques used to study the diffusion coefficients in the ECS have been discussed by Syková and Nicholson (2008).

#### 2.2.1. Boundary conditions

The boundary conditions at the boundaries of the brain with the needle, with the exception of the tip of the needle, as well as the outer boundaries of the brain are defined as “No Flux,” which means that no mass flows across the boundary

(4) $$-n\cdot \left(cu-D^{*}\nabla c\right)=0.$$

The boundary condition at the tip of the needle is defined as “Inflow,” where the concentration is set to a fixed value such that

(5) $$c=c_{0}.$$

Equations (3)–(5) form the governing equations of the system.

#### 2.2.2. Parameters

All simulation parameters are summarized in Table 1. The following paragraphs detail the derivation and source references for each of those parameters for dextran.

**Table 1:** **Solute drainage parameters used in the simulations**.

**Parameter**	**Value**	**Source**
Bulk flow velocity in white matter u	1.75 × 10^−7^ms^−1^	Rosenberg et al., 1980
Apparent perivascular drainage velocity u_*b*_	8.33 × 10^−6^ms^−1^	Carare et al., 2008
Diffusion coefficent D^*^	8.7 × 10^−11^m^2^s^−1^	Nicholson and Tao, 1993
Uptake rate *k*_*b*_	2.5 × 10^−4^s^−1^	Ito et al., 2013

As discussed above the mouse brain is simplified by homogenizing all gray and white matter (that is considering all gray and white matter as one). Accordingly, we have homogenized the tortuosity λ values reported in Syková and Nicholson (2008). Because of the sparsity of values for the mouse brain and the similarity of mouse and rat values we have used rat values where necessary. Note that tortuosity for the gray matter is isotropic, while tortuosity in the white matter is anisotropic due to the directionality of the fiber tracts, such that D^*^ becomes a matrix

(6) $$D^{*}=\left(\begin{array}{cc} D/\lambda_{x}^{2} & 0\\ 0 & D/\lambda_{y}^{2} \end{array}\right).$$

The tortuosity values used in this study are summarized in Table 2. The effective diffusion coefficient D^*^ for dextran (3 kDa) was obtained directly from Nicholson and Tao (1993), which has been measured at 34 °C. To correct this to a temperature of 37 °C we use

(7) $$D_{37}=\frac{T_{37}}{T_{20}}\frac{\mu_{20}}{\mu_{37}}D_{20},$$

where *T*_37_ and *T*_20_ are temperatures in *K*, μ_37_, and μ_20_ are the dynamic viscosities and *D*_37_ and *D*_20_ are the diffusion coefficients at temperatures *T*_37_ and *T*_20_, respectively. Using this equation and the dynamic viscosity of water (Philippoff, 1957) at the given temperatures we can correct our diffusion coefficient to D^*^ = 8.7 × 10^−11^ m^2^ s^−1^.

**Table 2:** **Tortuosity values for gray and white matter as obtained and summarized from (Syková and Nicholson, 2008)**.

**Region**	**λ_*x*_**	**λ_*y*_**	**λ_*z*_**
Gray matter	1.60	1.60	1.60
White matter	1.47	1.68	1.68

The importance of bulk flow along the white matter in the ECS is not clear and has been discussed in the literature (see for example Rosenberg et al., 1980; Ohata and Marmarou, 1992; Abbott, 2004). According to Syková and Nicholson (2008) the influence of bulk flow on short-term and near-distance diffusion is small, whereas it could have a significant impact over long distances and times. The influence of bulk flow, however, is hard to quantify. In this study we therefore implement simulations for both cases – with and without bulk flow along the white matter. Where a simulation includes bulk flow along the white matter we adopt the findings from Rosenberg et al. (1980) and assume a bulk flow velocity of |***u***| = 1.75 × 10^−7^ m s^−1^ following the direction of the fibers. We recognize that this value has been measured in the cat rather than the mouse, where the white matter is likely to be organized differently. However, to the best of our knowledge no other data on bulk flow along white matter are available in the literature and therefore we use the Rosenberg value. Recently, Iliff et al. (2012, 2013) have suggested a convective bulk flow pathway from arteries to veins in the ECS through the gray matter. However, this effect has not been quantified and has therefore not been included in this study.

Finally, the uptake term *kc* needs to be determined. The total uptake rate *k* determines the time scale at which tracers are eliminated from the ECS. Normally, this uptake rate encompasses two different mechanisms, which need to be considered separately. The first mechanism is the degradation of solutes by enzymes or cells at rate *k*_*d*_. As this mechanism is not relevant for dextran *k*_*d*_ has been eliminated. The second mechanism represents the transport of tracers across the blood-brain barrier (BBB) into the blood (Deane et al., 2004) and the transport into the perivascular drainage pathways within the basement membrane at rate *k*_*b*_. Note here that while we are using a first-order rate process to model the transport of tracers into the basement membrane this is not treated as a loss process. Instead, the term *k*_*b*_*c* appears as a source term in the equation governing the perivascular drainage mechanism. Ito et al. (2013) have measured both *k*_*d*_ and *k*_*b*_ for Aβ and we used their value for *k*_*b*_ = 2.5 × 10^−4^ s^−1^ for the uptake of dextran as we assume that the rate of molecule transport into the basement membrane is the same for Aβ and dextran. Furthermore, we assume that this process captures all first-order rate processes such that *k* = *k*_*b*_.

As in Carare et al. (2008) 0.5 µl of 3 kDa dextran at a concentration of 1 µg µl-1 was injected over a period of 2 min. To convert the units of concentration of dextran to molarity M, as is required for the chosen type of boundary condition in COMSOL, we calculate the molar mass of dextran by multiplying its molecular weight with Avogadro's constant. We can then divide the concentration given in µg µl-1 by the molar mass and obtain a molar concentration of *c*_0_ = 0.33 mM for the injection. The boundary condition (Equation 5) therefore becomes

(8) $$c=\{\begin{array}{ll} c_{0} & t=\;0-2\;\min\\ 0 & t>\;2\;\min \end{array}.$$

Additionally, one has to take into account the convective flow generated by the injection of tracer into the brain. We have conducted test simulations on the coronal slice using various convective flow velocities. These tests show that velocities of up 4.0 × 10-6 m s-1 (roughly half the perivascular drainage velocity) have a negligible effect on the tracer distribution. As we regard higher velocities than that too high given the time frame of the injection we have omitted this initial convective flow for the remaining simulations.

### 2.3. Perivascular drainage

Molecules “switch” from being in the ECS to being in the perivascular drainage pathways in the basement membranes of cerebral arteries at rate *k*_*b*_, which can be modeled in COMSOL using a “Reactions” node to account for production and consumption of chemical species. If we call the concentration of dextran in the ECS *c* and the concentration of dextran in the basement membranes *c*_*b*_, then *k*_*b*_ acts as a take-up coefficient and the term −*k*_*b*_*c* is part of Equation (3), while the equations for the transport of species in the basement membrane receive a source term *s*_*b*_ = *k*_*b*_*c*. Note that although we use different names for the concentrations of tracer in the ECS and the basement membrane these concentrations both describe dextran, but in different compartments. Note that in this representation we are modeling the two phenomena—parenchymal diffusion and perivascular convective flow—as two separate “layers” on the geometry. This means that every point in space is at the same parenchymal and perivascular.

As we assume that convective flow in the basement membrane is the dominant transport process we eliminate diffusion by setting the diffusion coefficient $D_{b}^{*}$ to the very small internal parameter “eps” in COMSOL, which represents the machine precision (it is not possible to set $D_{b}^{*}$ to 0 as this results in a singular matrix). The governing equation in the basement membrane is

(9) $$\frac{\partial c_{b}}{\partial t}=D_{b}^{*}\nabla^{2}c_{b}-(u_{b}\cdot \nabla )c_{b}+s_{b}.$$

#### 2.3.1. Boundary conditions

The boundary conditions are the same as for the ECS part of the simulation with the exception of the needle tip, which is changed to “No Flux” (Equation 4).

#### 2.3.2. Parameters

As the “production” of dextran in the basement membrane is directly coupled to the “loss” of dextran from the ECS the only parameter that remains to be determined is the perivascular drainage velocity ***u***_*b*_. As the lymphatic drainage pathways follow the basement membranes inside the walls of all arteries in the brain modeling these pathways explicitly and in detail is beyond the scope of this paper. Instead we can utilize measurements taken by Carare et al. (2008) about the distance dextran has traveled within the basement membrane. Two statements on the distance dextran has traveled can be found in Carare et al. (2008): (a) Dextran was found at a maximum distance of 2.5 mm from the injection site in the rostro-caudal direction after 5 min, which was measured by the number of 10 µm coronal slices from the injection site that still contained dextran, and (b) the dextran distribution had a mean radius of 785 µm. To model the observed perivascular drainage velocity we use statement (a) as statement (b) coincides with the observations when simulating diffusion only (see simulation case A1 in the Results section). This is valid as after 5 min the bulk of dextran is expected to still remain in the ECS. We therefore assume that it is the process of perivascular drainage that results in the observation of a maximum travel distance of 2.5 mm at 5 min. The easiest way of simulating a transport process that transports tracers to the given distance within the given time is by implementing its velocity as a vector ***u***_*b*_ = (*x, y*) = (8.33 × 10^−6^ m s^−1^, 0) in the coronal slice and ***u***_*b*_ = (*y, z*) = (0, 8.33 × 10^−6^ m s^−1^) in the sagittal slice.

This representation of the perivascular drainage pathways and their geometries is notably extremely simplified. However, a realistic representation could only be achieved through modeling of the whole vascular tree in the brain. For the aim of this paper, which is the comparison of the macroscopic phenomenon of dextran reaching 2.5 mm in 5 min to the effect of diffusion, our representation was deemed sufficient. Foley et al. (2012) have also measured convective flow velocity along perivascular pathways and it is interesting to note that they have measured velocities of one order of magnitude larger than our estimate. However, there are significant differences between their study and Carare et al. (2008), such as the use of nanoparticles whose molecular size is much larger than that of 3 kDa dextran. Therefore, we use the observation from Carare et al. (2008) as our perivascular drainage velocity estimate.

### 2.4. Simulation cases

All simulation cases are listed in Table 3. The Péclet number Pe is a dimensionless number to quantify the ratio of convective flow and diffusion and is defined as

(10) $$\text{Pe}=\frac{LU}{D},$$

where *L* is an appropriate length scale, *U* is velocity, and *D* is the diffusion coefficient. Here we choose the height of the mouse brain as the length scale. The Peclet number can be used to determine the balance between diffusion and advection that is required to obtain the expected results.

**Table 3:** **Simulation cases used to determine the necessity of convective transport mechansims to explain perivascular drainage**.

**Case**	Diffusion coefficient D^*^	**Bulk flow velocity in white matter u**	**Perivascular drainage velocity |u_*b*_|**	**Uptake rate *k*_*b*_**	**Pe**
A1	D^*^	0	0	0	0
A2	5·D^*^	0	0	0	0
A3	10·D^*^	0	0	0	0
B1	D^*^	*u*	0	0	11.08 (*u*)
B2	D^*^	5·u	0	0	55.38 (u)
B3	D^*^	10·u	0	0	110.76 (u)
C1	D^*^	u	u_*b*_	*k*_*b*_	527.22 (u_*b*_)
C2	D^*^	u	u_*b*_/2	*k*_*b*_	263.61 (u_*b*_)
C3	D^*^	u	u_*b*_/2	*k*_*b*_/10	263.61 (u_*b*_)
C4	5·D^*^	u	u_*b*_/2	*k*_*b*_/10	105.44 (u_*b*_)

D^*^ = 8.7 × 10^−11^ m^2^ s^−1^, |u| = 1.75 × 10^−7^ m s^−1^, |u_b_ = 8.33 × 10^−11^ m s^−1^, k_b_ = 2.5 × 10^−4^ s^−1^ (Table 1).

The goal of this paper is to show that diffusion alone is not enough to explain perivascular drainage. First, in cases A and B diffusion and bulk flow are considered as the only driving forces of solute drainage in the brain. The parameters for case C1 were determined from the results of the experiments in Carare et al. (2008), while the remaining simulation cases were designed to test reduced parameters for perivascular drainage against an elevated diffusion coefficient.

The simulation cases were set up to demonstrate that even if the reported values for diffusion and perivascular drainage have been under- or overestimated, respectively, diffusion alone remains unable to explain the experimental results from Carare et al. (2008). If in all of these simulation cases diffusion is unable to account for the experimental results in Carare et al. (2008) then we can claim with a high degree of confidence that a convective transport mechanism such as perivascular drainage must exist.

## 3. Results

To compare diffusion and perivascular drainage the maximum distance a tracer has traveled from the injection site is measured by calculating the largest distance between the injection site and a point whose tracer concentration is at least 0.1 % of the injection concentration. This dextran detection criterion has been established in pilot studies prior to Carare et al. (2008). The distance is calculated using a Euclidean distance measure

(11) $$d=\sqrt{{\left(x_{2}-x_{1}\right)}^{2}+{\left(y_{2}-y_{1}\right)}^{2}},$$

where *d* is the distance betweeen two points with the coordinates (*x*_1_, *y*_1_) and (*x*_2_, *y*_2_).

### 3.1. Coronal slice

The maximum distance for each of the simulation cases at both 5 and 30 min after the end of the injection is given in Table 4. Supplementary Figures 1, 2 show different levels of the distribution of dextran above the detection limit of 0.1 % of the injection concentration. The dextran concentration is non-dimensionalized using the minimum detection concentration.

**Table 4:** **Maximum spread of dextran in the coronal slice for each of the simulation cases at 5 and 30 min after the injection**.

**Case**	**Maximum distance from injection site at 5 min (mm)**	**Maximum distance from injection site at 30 min (mm)**
A1	0.76	1.35
A2	1.72	2.80
A3	2.20	3.38
B1	0.76	1.34
B2	0.74	1.41
B3	0.72	1.52
C1	2.46	2.46
C2	2.46	2.46
C3	0.76	2.46
C4	1.72	2.48

Cases A1–3 consider only diffusion as the driving force of solute transport. In none of the simulation cases does dextran reach as far as is expected from the results in Carare et al. (2008). If the actual diffusion coefficient is at least five times higher than the expected diffusion coefficient dextran can reach the surface of the brain within 30 min, but most dextran remains in the ECS at the injection site.

In cases B1–3 the effect of bulk flow is considered. Upon comparing these cases to A1 no signifcant effect of bulk flow along the white matter on solute transport is observed.

The effect of perivascular drainage is examined in cases C1–4. Only in case C1 does dextran appear to reach the distance of 2.5 mm within 5 min after the injection of dextran. However, the contours are disconnected. To aid the explanation of this in the discussion, Supplementary Figure 3 extends the first contour to 0.1, which represents a concentration of 0.01 % of the injection concentration. Cases C2–4 do not reach 2.5 mm within 5 min. In these cases diffusion appears to be the dominant process of solute transport until 5 min after the injection, when enough dextran has entered the perivascular drainage pathways in the basement membrane, while 30 min after the injection we observe the same results as for case C1.

**Figure 4:**
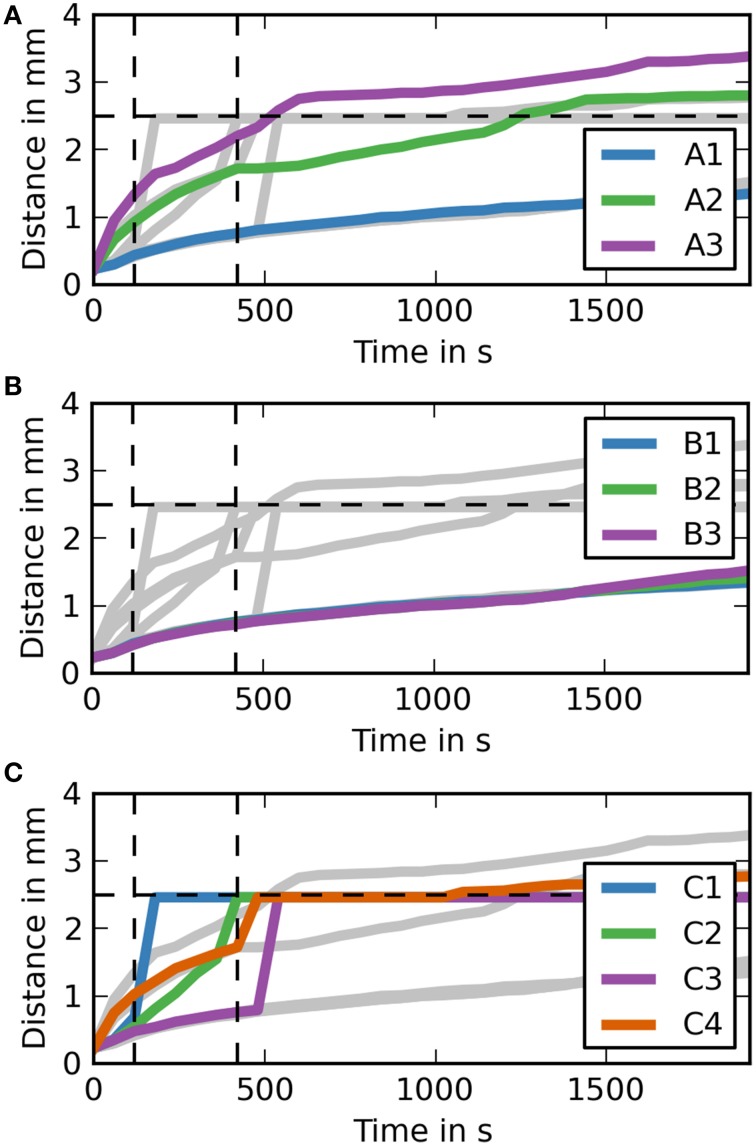
**Maximum distance dextran traveled for each simulation over time (coronal). (A)** The simulation cases A1–3 are highlighted against the other simulation cases. Dextran does not reach the expected distance in any of the simulation cases. It takes around 7.5 min after the injection for dextran to reach the 2.5 mm point in case A3, where the diffusion coefficient is increased by a factor of 10. **(B)** The simulation cases B1–3 are highlighted against the other simulation cases. The results for each of these simulations are very similar to case A1. **(C)** The simulation cases C1–4 are highlighted against the other simulation cases. In cases C1 and C2 dextran reaches the 2.5 mm point within 5 m after the injection, while the remaining two cases take up to 2 m longer. Case C4 takes longest to reach 2.5 mm and reaches it at the same time as the fastest diffusion case (A3).

In order to further analyse the distances dextran traveled in the different simulation cases it is useful to study the maximum distance dextran has traveled over time (Figure 4). Each of the subfigures shows the results for all simulation cases in the background in gray. Vertical dashed lines mark the time points 2 min into the simulation where the injection ends and 5 min after the end of the injection, while the horizontal dashed line marks the distance we expect dextran to have traveled at the 5 min time point. The results show that the only simulation cases reaching 2.5 mm are C1 and C2 and all simulation cases in group C are faster than all other simulation cases. None of the simulations in case group B reaches 2.5 mm at the end of the simulation time (30 min).

### 3.2. Sagittal slice

The sagittal slice produces very similar results compared to the results reported for the coronal slice. Table 5 shows the maximimum distance dextran has traveled at the 5 and 30 min time points. It shows that case C1 is the only case in which dextran spreads beyond 2.5 mm. In the coronal slice case C2 also reached the 2.5 mm mark.

**Table 5:** **Maximum spread of dextran in the sagittal slice for each of the simulation cases at 5 and 30 min after the injection**.

**Case**	**Maximum distance from injection site at 5 min (mm)**	**Maximum distance from injection site at 30 min (mm)**
A1	0.79	1.28
A2	1.75	2.95
A3	2.28	3.54
B1	0.78	1.29
B2	0.80	1.43
B3	0.94	1.57
C1	7.75	8.49
C2	1.40	8.06
C3	0.76	7.83
C4	1.73	8.16

Supplementary Figure 4 shows the contour levels of dextran concentration at 5 min after the injection (analogously to S1). Visual observation of these contour plots does not show dextran reaching a 2.5 mm distance in case C1. To aid the explanation of this observation a contour plot that includes the contour level of 0.1 is given in Supplementary Figure 3. Case C2 appears to reach around 2 mm. The results for the remaining cases are analogous to the respective cases in the coronal slice. The same holds for all cases in Supplementary Figure 5 (30 min after the injection).

**Figure 5:**
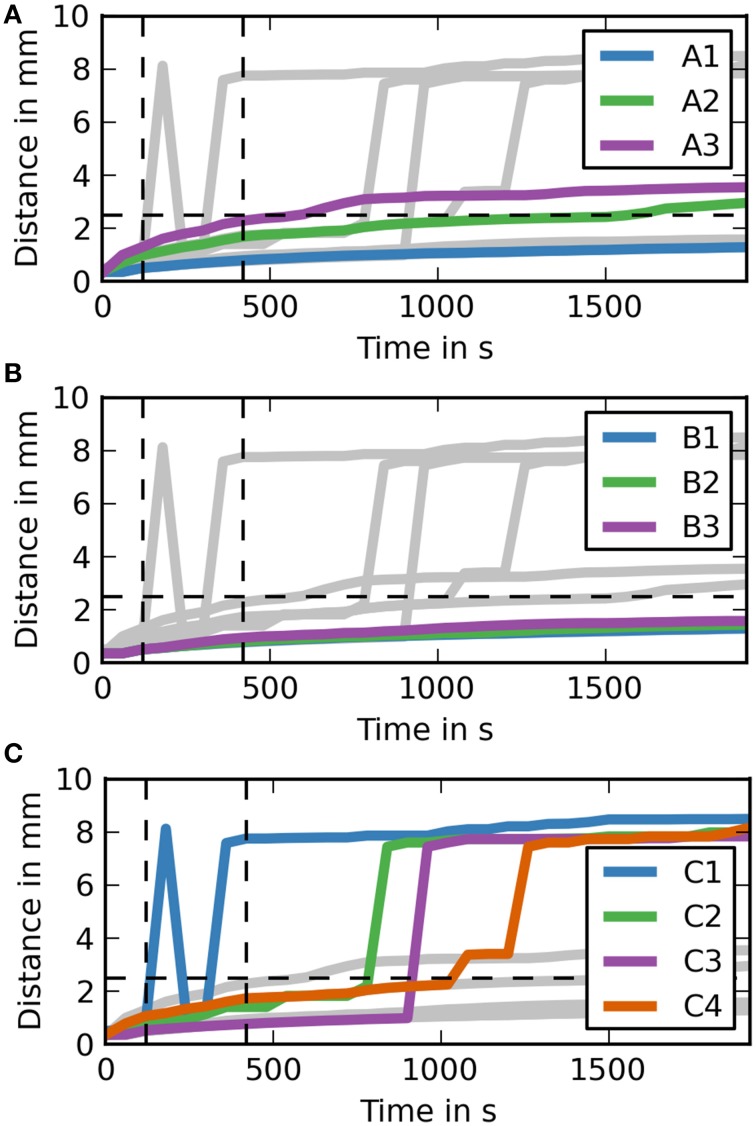
**Maximum distance dextran traveled for each simulation over time (sagittal). (A)** The simulation cases A1–3 are highlighted against the other simulation cases. Dextran does not reach the expected distance in any of the simulation cases. It takes around 7.5 min after the injection for dextran to reach the 2.5 mm point in case A3, where the diffusion coefficient is increased by a factor of 10. **(B)** The simulation cases B1–3 are highlighted against the other simulation cases. All of the cases are very similar to case A1. **(C)** The simulation cases C1–4 are highlighted against the other simulation cases. In two of them dextran reaches the 2.5 mm point within 5 min after the injection, while the remaining two cases take up to 2 min longer.

To aid the analysis of the results Figure 5 shows the maximum distance dextran has traveled from the injection site at any time during the simulation. For case groups A and B the results here are very similar again to the results of the coronal slice. The results for case group C are different. Case C1 gets an early spike at around 1 min after the end of the injection. This spike then drops back to the diffusion level, but then increases again to the required level before the 5 min time point.

## 4. Discussion

The results of this study demonstrate that diffusion alone cannot account for the pattern of drainage of fluid and solutes from the brain parenchyma. Our results show that complex convective transport pathways exist in the brain for the clearance of solutes produced during brain metabolism. The drainage pathway, therefore, is partly dependent upon diffusion through the ECS between cell processes in the brain parenchyma and partly reliant upon a more rapid system of convective flow along basement membranes within walls of cerebral capillaries and arteries.

The results of Supplementary Figure 1 match the results from Carare et al. (2008). None of the simulations in case group A (diffusion only) reach the 2.5 mm mark in 5 min after the end of the injection. Only case A3 gets close (2.2 mm) to the required distance mark, but this assumes that the diffusion coefficient reported in Nicholson and Tao (1993) is wrong by an order of magnitude. In case A1 dextran reaches a distance of 0.76 mm, which is close to the reported mean value of dextran distribution of 0.79 mm in Carare et al. (2008). The results of case A2 do not correlate with any of the results in Carare et al. (2008). At 30 min after the end of the injection (Supplementary Figure 2) both case A2 and A3 have traveled beyond the 2.5 mm mark while case A1 has not. This shows that the dextran found at the surface of the brain at 30 min after the end of the injection is a result of a convective transport mechanism. Figure 4, which shows the maximum distance dextran has traveled at any time compared to the remaining cases, confirms these results and also shows that the simulations from case group A can travel beyond the 2.5 mm mark. This is due to diffusion acting in all directions, whereas perivascular drainage is implemented to only act in the positive *x*-direction and therefore encounters the boundary of the brain at 2.46 mm.

For case group B the results presented in Supplementary Figures 1, 2 and Figure 4 show that bulk flow does not have a significant effect on solute drainage. All three cases in this group match the results of case A1 very closely for both time points and will therefore be omitted from the rest of the discussion.

In case group C both cases C1 and C2 manage to reach the distance mark of 2.5 mm within 5 min. In case C1, however, there are two separate disconnected sections of dextran concentration above the detection limit. To explain this phenomenon Supplementary Figure 3 includes a dextran concentration level of 0.1 (0.01 % of the initial concentration). It shows that dextran travels in a continuous stream to the surface of the brain. We observe two disconnected parts of dextran concentration because the stream between is below the detection level. In both cases C3 and C4 perivascular drainage is slowed down enough such that diffusion remains the dominant mechanism at that time point. This can also be seen in Figure 4 as up to 8 and 7 min, respectively, the curves of C3 and A1 and C4 and A2 are almost identical. At 30 min after the injection all cases in group C show a dextran concentration above the detection level at the surface of the brain (Supplementary Figure 1). While in case C4 this is due to the increased diffusion coefficient for cases C1–3 we observe the same disconnected smaller area of dextran as in case C1 at 5 min after the end of the injection. This shows that perivascular drainage has acted here as the main driving mechanism for solute drainage.

The simulations on the sagittal slice of the mouse brain have in general revealed very similar results as compared to the coronal slice (Supplementary Figures 4, 5 and Figure 5). The results for case group A are almost entirely identical, but dextran spreads slightly further within 5 min as Table 5 reveals. From Supplementary Figure 4 it appears that in case C1 dextran does not reach the 2.5 mm mark within 5 min. However, from both Table 5 and Figure 5 we can conclude that dextran does reach the distance mark within 5 min. To explain this Supplementary Figure 3 includes the concentration level 0.1. This figure reveals that in general dextran is spreading far away from the injection site and that C1 in Supplementary Figure 4 is similar to C1 in Supplementary Figure 5. In the sagittal slice, however, second area of dextran distribution above the detection limit is too small to observe in the figure. The results for the remaining cases of group C are analogous to the same cases in the coronal slice.

The Péclet numbers Pe of the simulation cases show that there appears to be a critical value, at which point the convective forces over the diffusive forces are strong enough to drive flow in the required time frame.

While the results of this study are in close agreement with our hypothesis of the necessity of perivascular drainage as the main mechanism for solute drainage in the brain there are some limitations to this study. The first limitation is the use of 2D simulations instead of 3D simulations. It was expected that, qualitatively, no additional information would be gained from using 3D over 2D simulations. This is confirmed by our results, which match those of the experiments conducted in Carare et al. (2008) very closely. Therefore, it was concluded that the advantages of requiring much smaller meshes for the simulations and therefore much less computation time weigh much more heavily against the most likely very small gain in accuracy of the results. Another limitation of this study is the implementation of the perivascular drainage process as a straight path in the positive *x*-direction for the coronal slice and in the positive *z*-direction for the sagittal slice. In reality the perivascular drainage follow the cerebral vasculature. To accurately represent perivascular drainage the whole cerebral vasculature would have to be modeled. As with the decision of using 2D over 3D simulations the decision of how to implement this process was made by looking at the information gain of a more complex model, which would require a much higher computational effort, compared to a simpler model. Based on this it was deemed legitimate to omit an explicit implementation of the perivascular drainage pathways and instead rely on this “observed velocity” along a straight line. Lastly we note that we have focussed exclusively on transport along basement membranes of capillaries and arteries as we study the clearance of solutes from the parenchyma. There is also a much wider field of study of how CSF communicates with the parenchyma and it remains to be seen to what extent the two systems interact with each other (Iliff et al., 2012, 2013).

## 5. Conclusion and relevance for Alzheimer's disease

We have established by a computational model that the rapid elimination of solutes from the central gray matter regions to the surface of the mouse brain is not due to diffusion alone but involves a combination of diffusion and bulk flow along basement membranes in the walls of capillaries and arteries. These results will help to establish the dynamics of periarterial lymphatic drainage that fails with age and AD. Furthermore, periarterial lymphatic drainage is relevant to recent trials of immunotherapy for AD in which insoluble plaques of Aβ are eliminated following the active or passive immunization of patients against Aβ (Nicoll et al., 2003). Despite removal of Aβ from brain parenchyma, however, there is a significant increase in the severity of CAA (Sakai et al., 2014), suggesting that Aβ is removed from the brain parenchyma but becomes entrapped in the ageing periarterial lymphatic drainage pathways. Whether this is due to the increased volume of Aβ passing into the lymphatic drainage pathways or whether it is due to the pathways themselves being blocked by immune complexes (Carare et al., 2013) has not been resolved. Understanding the dynamics of periarterial lymphatic drainage the brain will help to resolve questions related not only to the pathogenesis of AD, but also to questions related to immunotherapy.

## Author contributions

All authors have contributed to the manuscript preparation. The contributions are detailed below. AD: Manuscript preparation, development of simulations, and results analysis. MT: development of simulations. NB: development of simulations and results analysis. CH, AM: Manuscript preparation, particularly the introduction. RW, RC: Manuscript preparation, particularly introduction, and discussion.

## Funding

This work was supported by an EPSRC Doctoral Training Centre grant (EP/G03690X/1).

### Conflict of interest statement

The authors declare that the research was conducted in the absence of any commercial or financial relationships that could be construed as a potential conflict of interest.

## Supplementary material

Supplementary Figure 1 **Distribution of dextran 5 min after the injection (coronal slice)**. The concentration levels have been dedimensionalized using the minimum detection level of 0.1% of the injection concentration. Cases (A1–3) consider diffusion only with different diffusion coefficients. Cases (B1–3) consider the effect of bulk flow using the initial diffusion coefficent *D*^*^. Cases (C1–4) include a convective solute transport via perivascular drainage.

Supplementary Figure 2 **Distribution of dextran 30 min after the injection (coronal slice)**. The concentration levels have been dedimensionalized using the minimum detection level of 0.1% of the injection concentration. Cases (A1–3) consider diffusion only with different diffusion coefficients. Cases (B1–3) consider the effect of bulk flow using the initial diffusion coefficient *D*^*^. Cases (C1–4) include a convective solute transport via perivascular drainage.

Supplementary Figure 3 **Distribution of dextran 5 min after the injection (sagittal slice)**. The concentration levels have been dedimensionalized using the minimum detection level of 0.1% of the injection concentration. Cases (A1–3) consider diffusion only with different diffusion coefficients. Cases (B1–3) consider the effect of bulk flow using the initial diffusion coefficient *D*^*^. Cases (C1–4) include a convective solute transport via perivascular drainage.

Supplementary Figure 4 **Distribution of dextran 30 min after the injection (sagittal slice)**. The concentration levels have been dedimensionalized using the minimum detection level of 0.1% of the injection concentration. Cases (A1–3) consider diffusion only with different diffusion coefficients. Cases (B1–3) consider the effect of bulk flow using the initial diffusion coefficient *D*^*^. Cases (C1–4) include a convective solute transport via perivascular drainage.

Supplementary Figure 5 **Distribution of dextran 5 min after the injection in case C1 in both slices**. The lowest contour has been extended to include a level of 0.1, showing how dextran spreads in the brain.

## Author Note

All data supporting this study are openly available from the University of Southampton repository at http://dx.doi.org/10.5258/SOTON/386692.
