# Supplementary figures and images for: A Simulation Model of Periarterial Clearance of Amyloid-β from the Brain
Source: Front Aging Neurosci. 2016 Feb 12;8:18. doi: 10.3389/fnagi.2016.00018 (PMC4751273; doi:10.3389/fnagi.2016.00018)

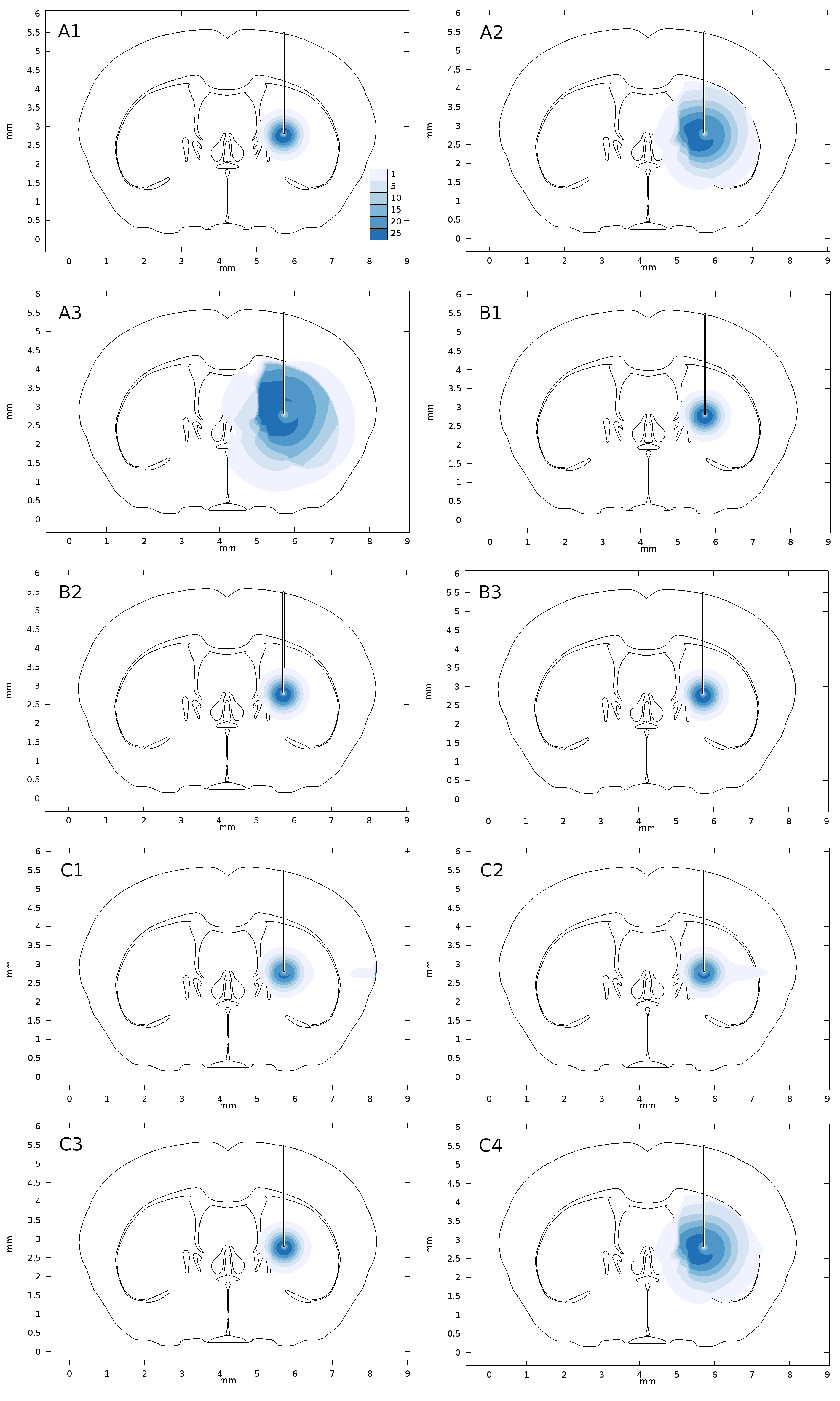

Supplement: Supplementary Figure 1 — Distribution of dextran 5 min after the injection (coronal slice). The concentration levels have been dedimensionalized using the minimum detection level of 0.1% of the injection concentration. Cases (A1–3) consider diffusion only with different diffusion coefficients. Cases (B1–3) consider the effect of bulk flow using the initial diffusion coefficent D*. Cases (C1–4) include a convective solute transport via perivascular drainage. [file Image1.PNG]

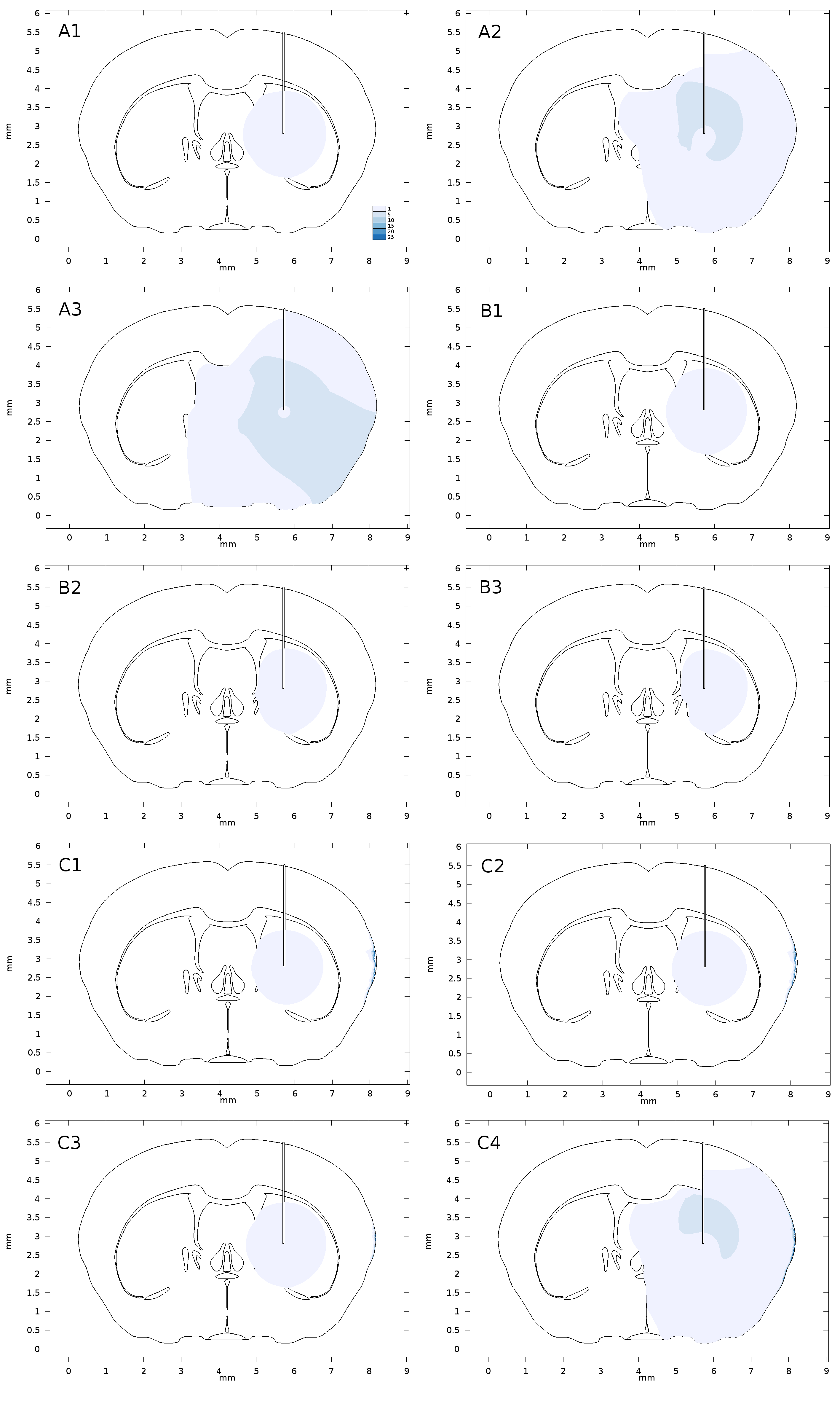

Supplement: Supplementary Figure 2 — Distribution of dextran 30 min after the injection (coronal slice). The concentration levels have been dedimensionalized using the minimum detection level of 0.1% of the injection concentration. Cases (A1–3) consider diffusion only with different diffusion coefficients. Cases (B1–3) consider the effect of bulk flow using the initial diffusion coefficient D*. Cases (C1–4) include a convective solute transport via perivascular drainage. [file Image2.PNG]

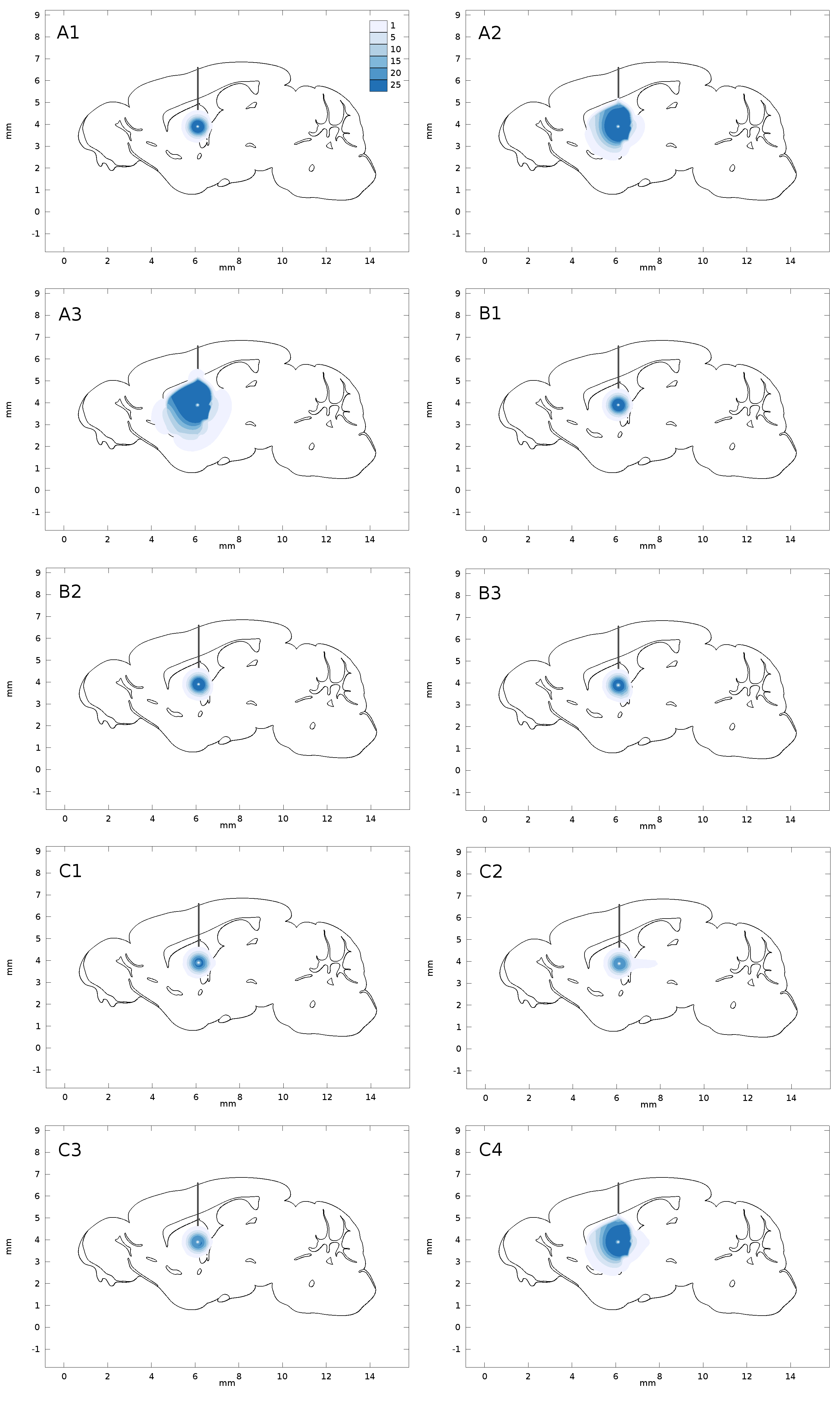

Supplement: Supplementary Figure 3 — Distribution of dextran 5 min after the injection (sagittal slice). The concentration levels have been dedimensionalized using the minimum detection level of 0.1% of the injection concentration. Cases (A1–3) consider diffusion only with different diffusion coefficients. Cases (B1–3) consider the effect of bulk flow using the initial diffusion coefficient D*. Cases (C1–4) include a convective solute transport via perivascular drainage. [file Image3.PNG]

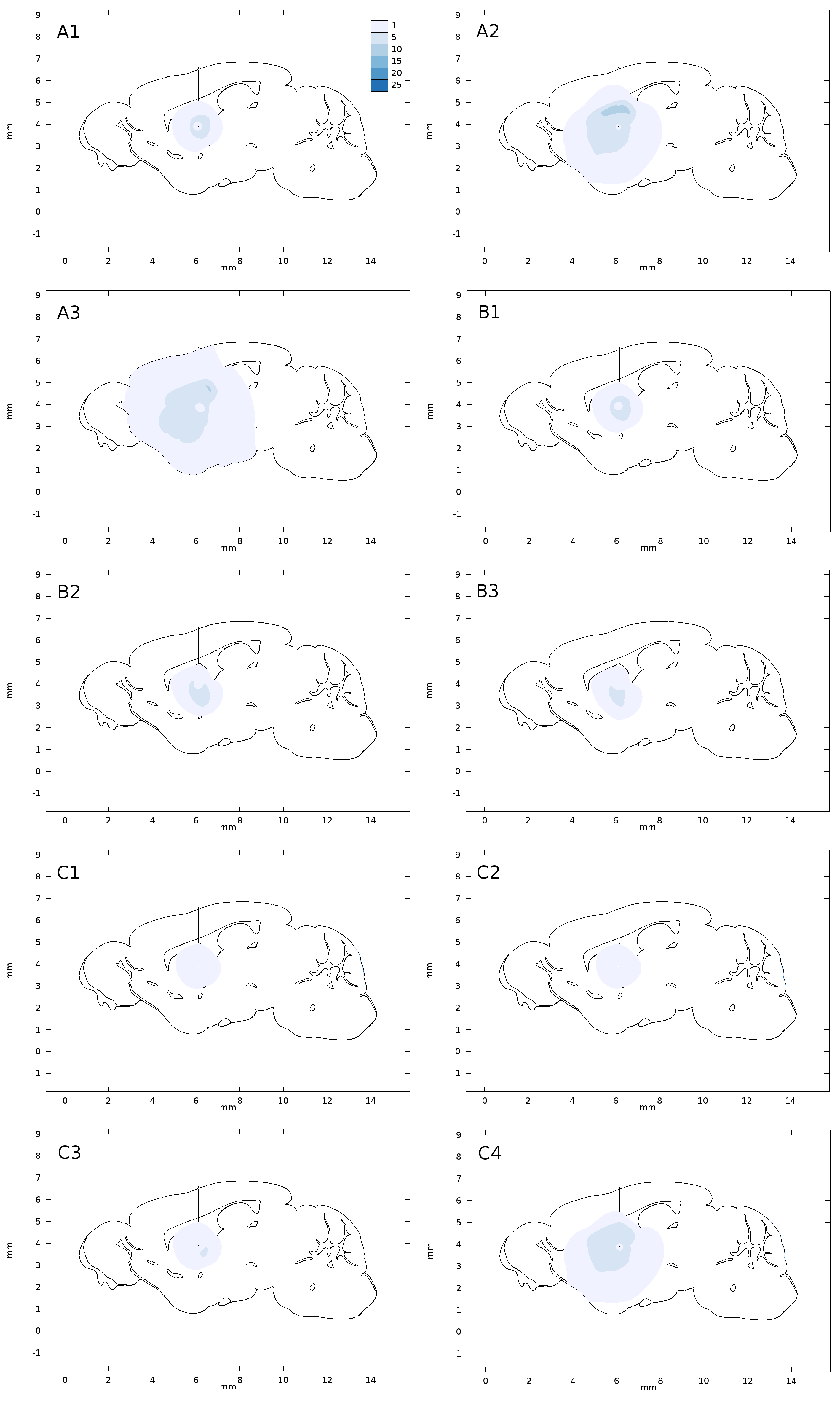

Supplement: Supplementary Figure 4 — Distribution of dextran 30 min after the injection (sagittal slice). The concentration levels have been dedimensionalized using the minimum detection level of 0.1% of the injection concentration. Cases (A1–3) consider diffusion only with different diffusion coefficients. Cases (B1–3) consider the effect of bulk flow using the initial diffusion coefficient D*. Cases (C1–4) include a convective solute transport via perivascular drainage. [file Image4.PNG]

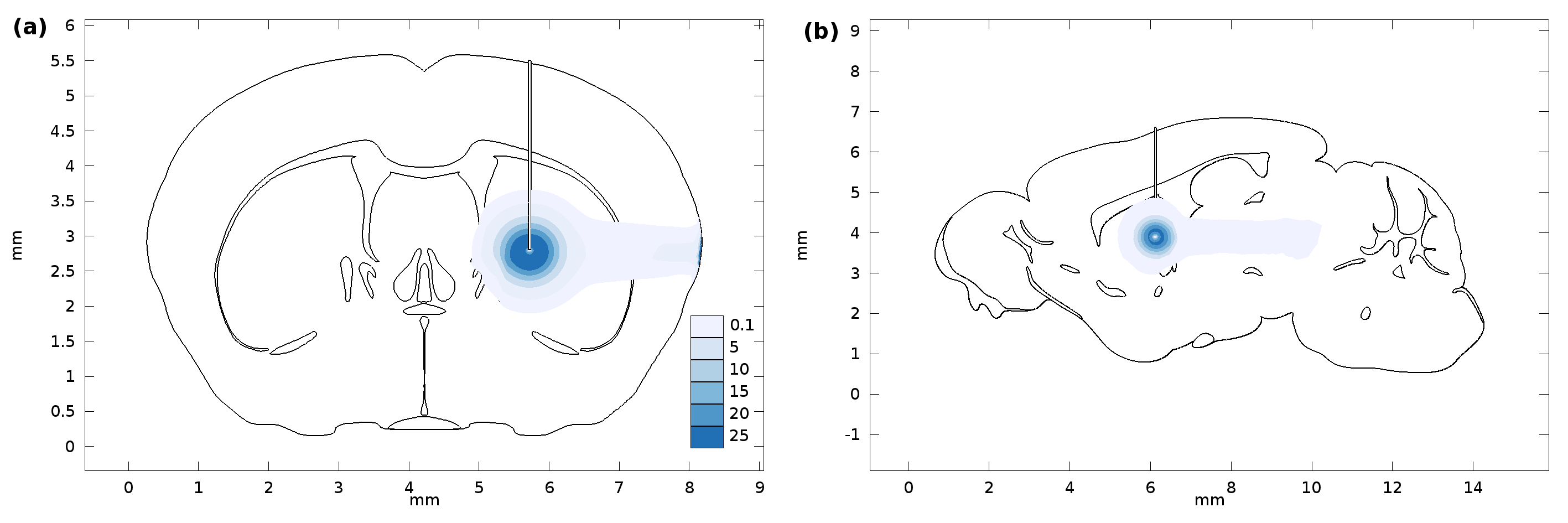

Supplement: Supplementary Figure 5 — Distribution of dextran 5 min after the injection in case C1 in both slices. The lowest contour has been extended to include a level of 0.1, showing how dextran spreads in the brain. [file Image5.PNG]
